# Mapping evidence on the distribution of human papillomavirus-related cancers in sub-Saharan Africa: scoping review protocol

**DOI:** 10.1186/s13643-017-0623-3

**Published:** 2017-11-17

**Authors:** Bridget K. M. Lekoane, Tivani P. Mashamba-Thompson, Themba G. Ginindza

**Affiliations:** Discipline of Public Health Medicine, School of Nursing and Public Health, University of KwaZulu-Natal, Mazisi Kunene Road, Durban, 4041 South Africa

**Keywords:** HPV-related cancers, Risk factors, Prevalence, Incidence, Mortality, Trends, HIV

## Abstract

**Background:**

Despite the introduction of HPV vaccines, the incidence of HPV-related cancers (cervical, penile, anal, vulvar, vagina, head, and neck) in sub-Saharan Africa has been rising. The increasing incidence of these HPV-related cancers has been attributed to changes in lifestyle-related risk factors, most notably sexual behavior. The main objective of this study is to map evidence on the distribution of HIV-related cancers in sub-Saharan Africa (SSA).

**Methods and analysis:**

We will conduct a scoping review to explore, describe, and map literature on the distribution of HPV-related cancers in sub-Saharan Africa. The primary search will include peer-reviewed and review articles. The list of references from included studies will also be searched. The search will be performed using EBSCOhost platform by searching the following databases within the platform: Academic search complete, health source: nursing/academic edition, CINAHL with full text, PubMed, Science Direct, Google scholar and World Health Organization (WHO) library databases, and gray literature. The researcher will search the articles using keywords, from the included studies; abstract and full articles will be screened by two independent reviewers. The screening will be guided by the inclusion and exclusion criteria. A thematic content analysis will be used to present the narrative account of the reviews, using NVivo version 10.

**Discussion:**

We anticipate finding relevant literature on the distribution of HPV-related cancers in sub-Saharan Africa. The study findings will help reveal research gaps to guide future research.

**Systematic review registration:**

PROSPERO CRD42017062403.

**Electronic supplementary material:**

The online version of this article (doi:10.1186/s13643-017-0623-3) contains supplementary material, which is available to authorized users.

## Background

The International Agency for Research on Cancer (IARC) defined human papilloma virus (HPV)-associated cancers as a specific cellular type of cancers diagnosed in a part of the body where HPV DNA is found [1]. HPV-related cancers are found on the parts of the body such as cervix, vulva, vagina, anal, penile, and oropharynx [2]. Per worldwide estimates, HPV infection is responsible for 5.2% of all cancers [3, 4]. Cancers associated with HPV are more prevalent in developing countries as compared to developed countries [5]. HPV is implicated in 99.7% of cervical cancer, 85% of anal cancers, 20% of oropharyngeal cancers, and 50% of cancers of the vulva, vagina, and penis cases worldwide [6]. According to estimates, cervical cancer is the fourth most common cancer among women with 572,624 new cases worldwide, which around 85% occurred in less developed regions. Around 266,000 females died of cervical cancer, accounting for 7.5% of all female cancer deaths [7].

WHO developed guidelines for prevention and control of cervical cancer; the main elements in the guideline were to vaccinate 9–13-year-old girls with two doses of HPV vaccine, use HPV tests to screen women for cervical cancer prevention, and communicate more widely to reach a wider audience e (http://www.who.int/reproductivehealth/RHUpdate/en/index.html). HPV vaccines are now broadly used in the prevention of cervical cancer; quadrivalent and bivalent are the two HPV vaccines developed towards mitigating the burden of cervical cancer. They have been pre-qualified by the World Health Organization (WHO) and approved by national government in many counties [8]. Also, the adoption of Papanicolaou(Pap) smear screening programs became successful and resulted in declining cervical cancer rates in Canada and the USA, but the incidence of other HPV-related cancers has been increasing [9, 10]. These increasing incidences of HPV-related cancers have been attributed to changes in lifestyle-related risk factors, most notably sexual behavior [10].

A scoping review of the literature regarding the distribution of HPV-related cancers in sub-Saharan Africa (SSA) is to be conducted. Interventions aimed at preventing and controlling cervical cancer in high endemic regions such as SSA are encouraged. However, research studies aimed at helping improve the efficiency of implementing these interventions are required. The aim of the study is to map evidence on the distribution of HPV-related cancers in sub-Saharan Africa. It is anticipated that the results of this study will reveal research gaps to guide future primary research and scoping reviews on HPV-related cancers in sub-Saharan Africa.

## Methods and materials

The current scoping review protocol is to be performed according to Preferred Reporting Items for Systematic Reviews and Meta-Analysis Protocols (PRISMA-P) guidelines [11] (Additional file 1: Figure S1), and the protocol is registered with PROSPERO under registration number CRD42017062403 and accessible via this link: https://www.crd.york.ac.uk/PROSPERO/display_record.asp?ID=CRD42017062403.

### Scoping review framework

The framework proposed review by Arksey H and O’Malley will be influential in conducting the scoping review [12]. The framework involves (I) identifying the research question, (II) identifying relevant studies, (III) study selection, (IV) charting the data, and (V) collating, summarizing, and reporting the results.

#### I. Identifying the research question

The major research question is “What is the evidence on the burden of HPV-related cancers in Sub-Saharan Africa?”

Research sub-questions are as follows:

- What is the burden of HPV-related cancers in sub-Saharan Africa with estimation on the prevalence, incidence, and mortality?
- What are the risk factors associated with HPV-related cancers?
- What are the trends of HPV-related cancers?
- What is the association of HIV and HPV-related cancers?

##### Eligibility criteria

Studies will be selected according to the PEO (population, exposure, and outcomes) framework outlined below (Table 1).

**Table 1:** PEO framework for eligibility of research question

Criteria	Determinants
Population and their problem	Individuals with HPV-related cancers in Sub-Saharan Africa
Exposure	HPV-related cancers
Outcome	1. Mortality
	2. Incidence
	3. Prevalence
	4. Risk factors(cervical cancer, anal cancer, penile cancer, vulva cancer, and head and neck cancers)
	5. Interplay with HIV 6. Trends

#### II. Identifying relevant studies

There will be no date and language restrictions applied on literature search. We will perform a keyword search from the following electronic databases: PubMed; World Health Organization (WHO) library; Science Direct; Google scholar; EBSCOhost platform for the following databases: Academic search complete, Health Source: Nursing/Academic Edition, and CINAHL with full text. We will use the following keywords while searching the above databases: HPV-related cancers, risk factors, prevalence, incidence, trends, HIV, and sub-Saharan Africa. Boolean terms AND and OR will be used to separate the keywords during the search. Mesh terms (Medical Subject Headings) will also be included in the search. The researcher will do a hand search of eligible studies from the list of references of included studies.

#### III. Study selection and eligibility

Following title screening from the above databases, articles with relevant title to the subject of the research will be uploaded on the Endnote X7 X7.7.1 software. Search results from different electronic databases will be combined in a single EndNote library (Appendix 1). Studies which do not address the research question and the duplicates of the same records will be excluded. From the included studies, abstract and full articles will be screened by two independent reviewers. An abstract screening form with questions will be developed based on the review eligibility criteria. The screening will be guided by the eligibility criteria. Discrepancies between reviewers at the abstract stage will be resolved through consensus and will involve a third reviewer. Discrepancies between reviewers at full article stage will be resolved by involving a third screener.

The relevant studies will be identified with guidance from the inclusion and exclusion criteria which were formulated according to the research questions.

##### Eligibility criteria

*Inclusion criteria*

- Study will include individuals with HPV-related cancers
- All study designs with relevant intervention
- Studies that focus on HPV-related cancers; prevalence, incidence, mortality, risk factors, and trends
- Studies that focus on HIV and HPV-related cancers.

*Exclusion criteria*

- Studies that do not focus on HPV-related cancers
- Studies that do not focus on humans
- Qualitative studies will also be excluded

PRISMA flow chart will be used to report the screening results.

Table 2 will be used to show the results of the titles searched from different databases.

**Table 2:** Database search record

Date of search	Search engine	Keyword searched	Number of articles found	Number articles eligible

PRISMA flow chart (Additional file 1: Figure S1) will be used to summarize the inclusion and exclusion criteria.

#### IV. Charting the data

Data from included studies will be extracted using an extraction form (Appendix 2). A data charting form will be developed and will be used to determine which variables to extract that will help to answer the research question. The extraction form will continually be updated. The form will include the following: author with date, study title, study design, study setting, population, study aim, intervention, percentages, outcomes of the study, key findings, and comments (Table 3).

**Table 3:** Data charting form

Author and date	
Study design	
Study setting	
Population	
- Average age	
- Sample size	
Aims	
Intervention	
Outcome	
Key findings	
Conclusion	
Comment	

#### V. Collating, summarizing, and reporting the results.

The aim of this study is to map the existing evidence and to summarize the findings as presented in articles. A narrative account of findings from existing literature will be presented through thematic content analysis. Literature will be extracted and structured around the following outcomes: prevalence, incidence, mortality, risk factors, trends, and HIV. The resulting themes will be analyzed and results will inspect the relationship between the findings and the research question. The meaning of the findings will be considered as they relate to the overall study purpose and the implications of these findings for future research, policy, and practice.

### Quality assessment

The quality of the evidence obtained from the studies will be assessed to make sure the study design is appropriate for the research objectives and the studies are reported well and to eliminate risk of bias. A quality appraisal tool which focuses on the study methods, the Mixed Method Quality Appraisal Tool (MMAT) Version 2011, will be used [13]. The tool will be used to examine the quality of an article looking at the following aspects: the appropriateness of the aim of the study, adequacy and methodology, study design, participant recruitment, data collection, data analysis, presentation of findings, authors’ discussions, and conclusions.

## Discussion

The current scoping review aims to map available evidence regarding the distribution of HPV-related cancers, the risk factors, and its association with HIV and AIDS in sub-Saharan Africa in order to reveal research gaps in this area. High-risk HPV types are the cause of all cervical cancers, anogenital cancers including the vulva, and anal and penile cancers [14, 15], as well as head and neck cancers [16]. Sub-Saharan Africa has the highest incidence of HPV and cervical cancer in the world [17]. HPV-related cancers are rising and they are a major public health concern exacerbating current disease burden in sub-Saharan Africa [18]. The burden of HPV-associated diseases is substantially increased where there is a HIV-1 co-infection [19], and with diverse HIV/AIDS epidemic in sub-Saharan Africa, the burden of HPV-related cancers might still rise. The Sustainable Development Goals (SDG) include targets relevant to women’s cancers, including a one-third reduction in premature mortality from non-communicable diseases through prevention and treatment by 2030 and achievement of universal health coverage, with access to quality essential health-care services and access to safe, effective, quality, and affordable essential medicines and vaccines for all [20].

The focus of this review is on HPV-related cancers, hence excluding studies that do no focus on HPV-related cancers and humans as the data will be irrelevant and will not report according to the set research questions. The study will focus on all individuals with HPV-related cancers regardless of gender. There is a latency period of 10 years or more between the initial high-risk HPV infection and the development of cancer; hence, there are no date restrictions on the literature search. Since the study is aimed on mapping the distribution of the HPV-related cancer, qualitative studies will be excluded in this review.

Despite information about HPV vaccine and testing within cervical cancer screening programs, it has been shown that patients’ and public’s knowledge of HPV-related cancers is still very little [21, 22]. Therefore, the results of this study will help address this knowledge gap. It is anticipated that our study findings will help to strengthen a need for policy implementation for prevention of HPV-related cancers and raise more awareness of HPV-related cancers. Also, government and stakeholders can be able to ensure that HPV vaccines are introduced everywhere as part of a coordinated and comprehensive strategy to prevent cervical cancer and other diseases caused by HPV. There will also be proper implementation of education on reducing behaviors that increase the risk of acquiring HPV infection, training of health workers and information to women about screening, diagnosis, and treatment of precancerous lesions and cancer especially in underdeveloped and financially constrained countries in sub-Saharan Africa. The finding will also help strengthen the need of HPV vaccines to be included in national immunization programs in the whole of sub-Saharan Africa.

## Appendix1

**Table 4:** How results from databases will be recorded

Date of search	Search engine used	Keyword search	Number of publications retrieved
18/07/2017	PubMed	((“epidemiology”[Subheading] OR “epidemiology”[All Fields] OR “prevalence”[All Fields] OR “prevalence”[MeSH Terms]) AND hpv[All Fields] AND related[All Fields] AND (“neoplasms”[MeSH Terms] OR “neoplasms”[All Fields] OR “cancers”[All Fields])) AND (“loattrfull text”[sb] AND “humans”[MeSH Terms])	1268

## Appendix2

**Table 5:** Sample data extraction form

Study details	ᅟ
Author/year	ᅟ
Objectives	ᅟ
Participants (characteristics/total number)	ᅟ
Setting/context	ᅟ
Interventions	ᅟ
Search details	ᅟ
Sources searched	
Years of included studies	
Number of studies included/types of studies included	
Country of origin of included studies	
Appraisal	ᅟ
Appraisal instruments used	
Appraisal rating	
Analysis	ᅟ
Method of analysis	
Outcome assessed	ᅟ
Results/findings	ᅟ
Significance/direction	ᅟ

## Additional file

Additional file 1: Figure S1. PRISMA flow diagram. (DOCX 33 kb)

## Abbreviations

AIDS: Acquired immunodeficiency syndrome
DNA: Deoxyribonucleic acid
HIV: Human immunodeficiency virus
HPV: Human papilloma virus
IARC: International Agency for Research on Cancer
MMAT: Mixed Methods Appraisal Tool
PRISMA-P: Preferred Reporting Items for Systematic Reviews and Meta-Analysis Protocols
SDG: Sustainable Development Goals
SSA: Sub-Saharan Africa
WHO: World Health Organization
