# Supplementary material for: Mapping evidence on the distribution of human papillomavirus-related cancers in sub-Saharan Africa: scoping review protocol
Source: Syst Rev. 2017 Nov 17;6:229. doi: 10.1186/s13643-017-0623-3 (PMC5693799; doi:10.1186/s13643-017-0623-3)
Supplement: Additional file 1: Figure S1. — PRISMA flow diagram. (DOCX 33 kb) [file 13643_2017_623_MOESM1_ESM.docx]

**ADDITIONAL FILE 1**

**Figure 1: PRISMA Flow Diagram**

## Screening

## Eligibility

Records identified through database searching
(n =)

Additional records identified through other sources
(n =)

Records after duplicates removed
(n =)

Records screened
(n =)

Records excluded
(n =)

Full-text articles assessed for eligibility
(n =)

Full-text articles excluded, with reasons
(n =)

## Identification

## Included

Studies included in qualitative synthesis and thematic content analyses
(n =)
